# Clinical Study on the Effectiveness of Three Products in the Treatment of Herpes Simplex Labialis

**DOI:** 10.1038/s41598-020-63530-6

**Published:** 2020-04-15

**Authors:** Hanna Boes, Vlasios Goulioumis, Anna Wechsler, Stefan Zimmer, Mozhgan Bizhang

**Affiliations:** Department of Operative and Preventive Dentistry, Faculty of Health, Witten/Herdecke University, Witten, Germany

**Keywords:** Oral herpes, Oral medicine, Oral pathology

## Abstract

Herpes simplex labialis (HSL) is a viral disease that affects the perioral region. No guidelines recommending an effective treatment exist. The treatment of HSL with three different products was examined. Herpatch Serum, a film-forming patch, was compared to Compeed Patches, a set of semiocclusive hydrocolloid patches, and Zovirax Cream (ingredient: 5% acyclovir). In this prospective, randomized, examiner-blind study, 180 patients with recurrent HSL were split into three groups (Compeed: *n* = 60, Herpatch: *n* = 60, Zovirax: *n* = 60) and examined within 24 hours of HSL outbreak (DRKS Registration No.: DRKS00007786). The primary endpoint was healing time. The secondary endpoints were the reaction rate and quality of therapy evaluated by the Clinician’s Global Assessment of Therapy (CGAT) and the Subject’s Global Assessment of Therapy (SGAT) (0 = no response; 10 = excellent response), respectively. There was no significant difference among the healing times for the different products. The mean (95% confidence interval) was 9.67 days (9.11–10.22) for Compeed, 9.30 days (8.75–9.85) for Herpatch, and 9.80 days (9.30–10.30) for Zovirax. The reaction rate and quality of therapy (CGAT and SGAT) of Herpatch were significantly higher than those of Compeed and Zovirax. Within the study limitations, Herpatch proved to be an effective, non-antiviral alternative in the treatment of HSL.

## Introduction

Herpes simplex labialis (HSL) is a worldwide infection of the oral and perioral regions caused by an infection with Herpes Simplex Virus type 1 (HSV-1) or, rarely, Herpes Simplex Virus type 2 (HSV-2). Despite the high prevalence of HSL, the disease has not yet been well characterized. The seroprevalence of HSV-1 in Germany was 78.4% from 2008 until 2011^1,2^. While many infections remain subclinical, other patients suffer from painful recurrent infections.

Following the primary infection with HSV-1, the virus replicates at the site of infection and proceeds to travel retrograde down unmyelinated sensory fibres to the trigeminal ganglion, where it establishes latency^3^. Interfering triggers such as psychological or physiological stress, UVB radiation, trauma, menstruation, sideropenia or febrile illness can provoke the reactivation of the virus^4,5^.

The course of disease in recurrent HSL generally involves several stages. Within the precursor stage, pain, tingling, or burning sensations in the affected area may occur. This is followed by the appearance of a macule, turning into a papule and leading to the formation of a vesicle. The vesicle bursts, and the formation of a soft scab is initiated. Subsequently, the soft scab is replaced by a hard scab. Over time, the scab falls off, and the lesion is fully healed without scarring^6–8^. While healing, the process is often accompanied by symptoms such as pain, discomfort or reduced self-esteem^6^. Complete healing takes seven to ten days^7^.

The treatment of recurrent HSL turns out to be very difficult and variable. Because of the slight effects of diverse therapies, the infection represents a constant and global public health problem^9^. Episodic therapy can be used for relief of accompanying symptoms^10^. Therefore, several topical applications, such as phytotherapeuticals, natural remedies, photodynamic therapy, heat and laser application, as well as antiviral substances, can be provided^11–16^.

The application of antiviral creams such as the Zovirax Cream (Zovirax; Glaxo Wellcome Operations, Barnard Castle, England), is favoured by most patients. Zovirax includes 5% acyclovir as an active ingredient. Acyclovir enters the affected cells and represses viral replication. The cream soothes symptoms such as pain, itching and burning. It is applied five times daily on the lesion and adjoining skin until complete healing occurs. Application should occur on at least four consecutive days but should not exceed 10 days^17^. Due to the potential for irritation and contact sensitization, the cream should not be applied on the skin if hypersensitivities against the components exist. Systemic toxicology studies were not required since an overdosage of the cream is unlikely because of the minimal exposure^18^.

An alternative is provided in the Compeed Invisible Cold Sore Patches that include no antiviral ingredient (Compeed; Johnson & Johnson Santé Beauté France SAS, Sézanne, France). The cytotoxicity was tested on fibroblasts. Further studies examined the irritation of the patches on rabbits and the sensitivity on guinea pigs. The outer layer of the patch consists of a semiocclusive polyurethane film, which allows excessive wound exudate to evaporate while keeping the lesion moist, ensuring an optimum moisture level. The patch seals the wound and arranges ideal conditions for wound healing. The patch functions as a shield, protecting surrounding areas from infection. As soon as the first symptoms occur, the patch is placed on the lesion, replaced if required, and worn for 24 hours until complete healing occurs^19^.

The newly developed Herpatch Serum (Herpatch; Sylphar NV, Deurle, Belgium) is spread on the lesion after the first symptoms of HSL occur, and within a few moments, it forms a transparent physical protective barrier over and around the lesion. Thus, the serum creates a microenvironment that provides optimal conditions for natural healing. Added ingredients such as zinc sulfate, porphyridium polysaccharide, and beta-glucan improve and accelerate wound healing, protect the lesion from drying out and UV radiation, relieve symptoms, strengthen the skin layers, and improve aesthetics^20^. The toxicological characteristics of the ingredients present in Herpatch suggest no important toxicological effects. Therefore, the serum is considered safe in cases of swallowing and with regard to topical absorption^21^. Since Herpatch does not include antiviral substances, it presents a potential alternative in the treatment of HSL. To date, Herpatch has not been compared to other products regarding effectiveness. For this reason, Herpatch was compared to Compeed and Zovirax in the following trial.

## Methods

### Study design and study population

This monocentre study is a prospective, controlled, randomized assessor-blind trial comparing the effectiveness of three products in the treatment of HSL. A total of 180 patients aged between 18 and 65 years with recurrent HSL participated. Within the first 24 hours of HSL outbreak, the patients visited the dental clinic of the University of Witten/Herdecke. The lesion had to present itself as a precursor lesion, macule, papule, vesicle or ulcer. Data were collected from April 2013 until June 2016. Informed consent was obtained from all 180 patients included in this study.

The subjects participating in this study needed to be between 18 and 65 years and were excluded if they showed any painful illnesses of the teeth or gingiva. Most importantly, the outbreak of the HSL could not have occurred more than 24 hours ago.

Subjects were excluded if they showed limited compliance, suffered from severe illnesses, or had taken antibiotics or anti-inflammatory drugs two weeks ahead of participation. Pregnant or breastfeeding females were excluded. Hypersensitivity against the ingredients contained in the products or alcohol or drug abuse were additional exclusion criteria. If the patient had already applied a product for the treatment of HSL on the present lesion or had participated in a different clinical study, the subject was not allowed to participate.

Before the beginning of the trial, the Independent Ethics Committee of the University of Witten/Herdecke permitted (written consent) the execution of this study (Application-No. 94/2012). Additionally, the study was registered at the German Clinical Trials Register (DRKS-No.: DRKS00007786; Registration-Date: 16. June 2015). The methods in this trial were performed in accordance with the ethical standards laid down in the 1964 Declaration of Helsinki for medical research involving humans and its later amendments as well as the principles of Good Clinical Practice.

### Study assessment

Throughout the study, several data points were collected by the clinician and the subject. On the initial examination day (day 1), the name, age, and sex of the patient were documented as well as the date and time of the beginning of the trial. Additionally, the characteristics of the HSL lesion were noted: site (upper or lower red of the lip, left or right corner of the mouth, underneath or above the lip), maximum diameter, and lesion stage.

The lesion stage (precursor lesion, macule, papule, vesicle, ulcer, soft scab, hard scab) was classified by the clinician on each examination day. At the end of the observation period (day 10), the clinician, using the Clinician’s Global Assessment of Therapy (CGAT) evaluated the reaction rate (assessment of the speed of effectiveness of the product) and the reaction quality (assessment of the effectiveness of the product). This assessment was taken over from a clinical trial by Karlsmark *et al*.^22^.

The subject was accompanied by a patient’s questionnaire that was filled out on each examination day, assessing the following parameters: severity of symptoms (pain, discomfort, itching, burning, tingling, swelling, soreness, vesicle, scab), protection of the lesion, aesthetics, relief of discomfort, and intolerance. At the end of the observation period (day 10), the subject also assessed the reaction rate and reaction quality using the Subject’s Global Assessment of Therapy (SGAT). Additionally, he or she rated the comfort, functionality, handling, and satisfaction of the applied product (Table 1).

**Table 1:** Assessed data by the clinician and subject during the trial.

Examination day	Day 1	Day 2	Day 4	Day 6	Day 8	Day 10
**General information**						
Personal data (name, age, sex)	X					
Inclusion/exclusion criteria	X					
Time of beginning of the trial	X					
Assignment of screening number	X					
Agreement of participation	X					
**Characteristics of HSL lesion**						
Site	X					
Diameter of lesion (cm)	X					
Photo documentation	X					X
Lesion/healing stage	X	X	X	X	X	X
**Clinician’s Global Assessment of Therapy**						
Reaction rate						X
Reaction quality						X
**Subject’s Global Assessment of Therapy**						
Occurring symptoms (pain, discomfort, itching, burning, tingling, swelling, soreness, vesicle, scab)	X	X	X	X	X	X
Protection of lesion	X	X	X	X	X	X
Aesthetics	X	X	X	X	X	X
Relief of discomfort	X	X	X	X	X	X
Tolerance	X	X	X	X	X	X
Comfort						X
Functionality						X
Handling						X
Satisfaction						X
Reaction rate						X
Reaction quality						X

### Treatment regimen

To ensure that the assessor was properly blinded, the products were randomly packed into nontransparent envelopes. In chronological order, the envelopes were handed out to the subjects.

Within 24 hours of the outbreak of the HSL, the patients were examined, and therapy with the assigned medication was initiated. Subjects were instructed to use the products as recommended on the package insert. Treatment was finished when the lesion was healed or after 10 days, whichever came first.

Participation in this study lasted 10 days. On the first day of participation, the subject attended the dental clinic within the first 24 hours of the outbreak of HSL. The inclusion and exclusion criteria were checked. If the patient fulfilled the inclusion criteria, he or she was given a detailed explanation of the reason for and process of the trial. Subsequently, the HSL lesion was documented by taking a photo, noting the stage and site, and measuring the maximum diameter. The patient’s questionnaire and the envelope with the medication were handed out to the participant.

On the 2^nd^, 4^th^, 6^th^, 8^th^ and 10^th^ days, the subject returned at the clinic, and the present stage of the lesion was documented. On the last examination day, the clinician documented the present situation by taking a photo.

### Outcome measures

The primary endpoint examines the existence of differences between the three products in terms of healing time. The assessments of the reaction rate and the reaction quality by the clinician (CGAT) and by the subject (SGAT) were defined as the secondary endpoints. The analysis of the patient’s questionnaire was determined as the tertiary endpoint.

### Statistical analysis

To produce significant results, the sample size was estimated with the programme G*Power 3.1.9.2^23^. A sample size of 180 patients (60 participants per treatment group) was calculated with a statistical power of 0.8, an alpha error of 0.05, and a 15% drop-out quote with an effect size of 0.5. All data were statistically analysed with the program SPSS 24.0.

The survival curves of the healing time for the primary endpoint were determined using the Kaplan-Meier estimator, and comparisons among the three different treatments were performed by the log-rank test (*p* < 0.05). All data are fully available without restriction.

The Kolmogorov-Smirnov, Kruskal-Wallis, and Mann-Whitney U tests were used to determine the existence of statistically significant differences among the three tested products regarding the secondary endpoint. After Bonferroni correction, the probability of error *p* and the level of significance were defined as *p* = 0.025.

The primary and secondary endpoints were statistically analysed. The tertiary endpoint was analysed descriptively to characterize and provide insight into the overall treatment effects.

## Results

### Characteristics of study participants and lesions

**Figure 1:**
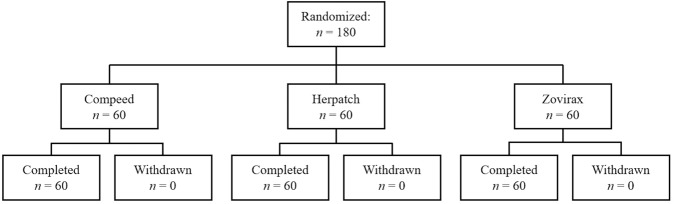
Subject allocation.

A total of 180 subjects participated and met the inclusion criteria. There was no drop-out (Fig. 1).

Overall, 81.67% of the subjects were females, and the mean age was 31.46 years (SD: ± 10.79; range: 18–61 years). At the onset, the sizes of the lesion were not significantly different among the three groups. The mean diameter was 0.65 cm. Most of the lesions were located on the upper and lower red of the lip. The locations of the sites showed a similar distribution in all three groups (Table 2).

**Table 2:** Demographic characteristics and characteristics of HSL lesions.

Product	Compeed (n = 60)	Herpatch (n = 60)	Zovirax (n = 60)
**Demographic characteristics**			
Sex, n (%)			
Male	9 (15.00%)	10 (16.67%)	14 (23.33%)
Female	51 (85.00%)	50 (83.33%)	46 (76.67%)
**Age, y**			
Mean (SD)	32.42 (11.50)	31.42 (10.36)	30.55 (10.57)
**Characteristics of HSL lesion**			
Diameter, cm			
Mean (SD)	0.70 (0.29)	0.60 (0.32)	0.66 (0.30)
**Site, n (%)**			
Upper red of the lip	22 (36.67%)	27 (45.00%)	29 (48.33%)
Lower red of the lip	22 (36.67%)	20 (33.33%)	14 (23.33%)
Right corner of the mouth	3 (5.00%)	2 (3.33%)	1 (1.67%)
Left corner of the mouth	4 (6.67%)	1 (1.67%)	2 (3.33%)
Underneath the lower lip	4 (6.67%)	5 (8.33%)	5 (8.33%)
Above the upper lip	5 (8.33%)	5 (8.33%)	9 (15.00%)

### Healing time

**Figure 2:**
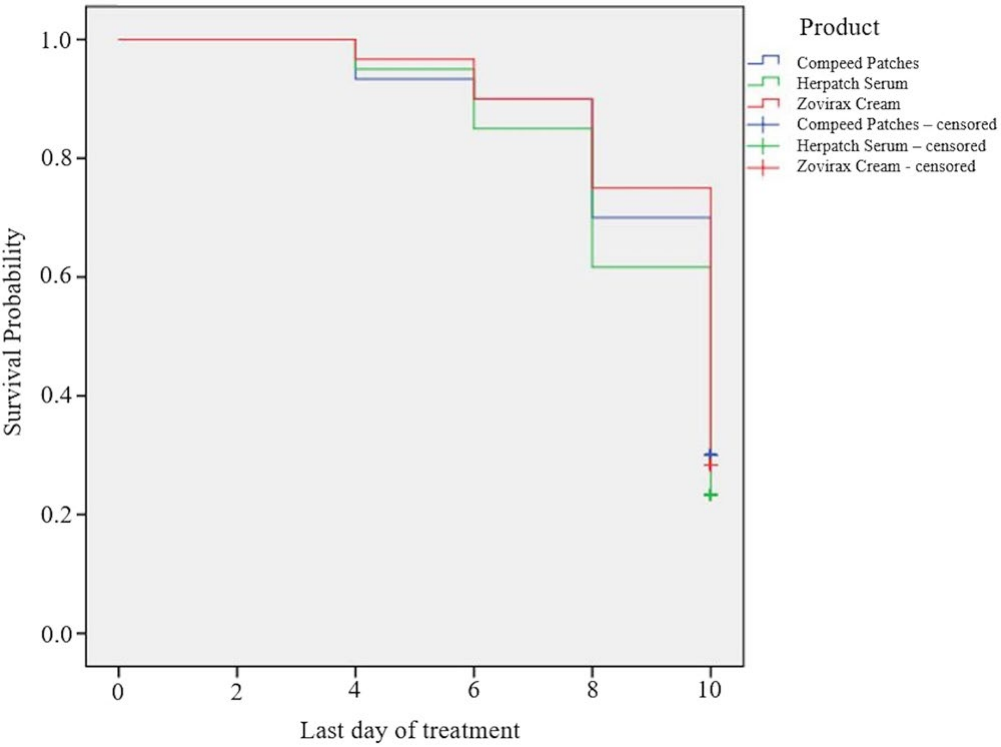
Kaplan-Meier curves for the healing time of HSL episodes during the 10-day investigation with each of the three products (log-rank test, no statistically significant differences among the three groups).

There was no statistically significant difference (log-rank test; *p* = 0.414) in the healing time among the three products. The mean (95% confidence interval) healing time was 9.67 days (9.11–10.22) for Compeed, 9.30 days (8.75–9.85) for Herpatch and 9.80 days (9.30–10.30) for Zovirax (Fig. 2).

### Reaction rate and reaction quality

The reaction rate and reaction quality were evaluated at the end of the therapy by the clinician (CGAT) and the subjects (SGAT) on a scale from 0 to 10 (0 = no response; 10 = excellent response). Both parameters, evaluated by the clinician and subject, were significantly higher for Herpatch compared to Compeed and Zovirax.

**Figure 3:**
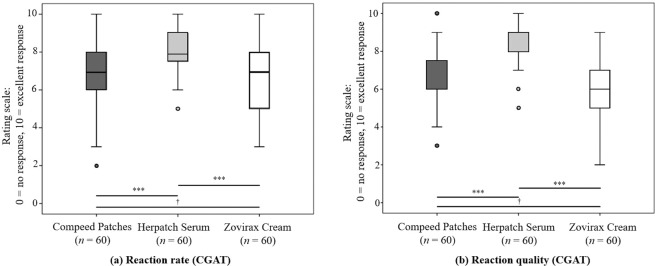
Median (25th and 75th percentile), minimum and maximum of the reaction rate (**a**) and reaction quality (**b**) of the product assessed by the clinician (CGAT) at the end of therapy (day 10). Horizontal lines with “***” indicate significant differences between the products, and “†” indicates the lack of a statistically significant difference.

The median (25th and 75th percentile) reaction rate (CGAT) of Herpatch was 8.00 (7.25–9.00) and was significantly different (*p* = 0.001) from that of Compeed, with a median of 7.00 (6.00–8.00), and that of Zovirax, with a median of 7.00 (5.00–8.00). Additionally, the median (25th and 75th percentile) reaction quality (CGAT) showed significantly greater (*p* = 0.001) for Herpatch, with a median of 8.00 (8.00–9.00), than for Compeed, with a median of 7.00 (6.00–8.00), and for Zovirax, with a median of 7.00 (5.00–8.00) (Fig. 3).

**Figure 4:**
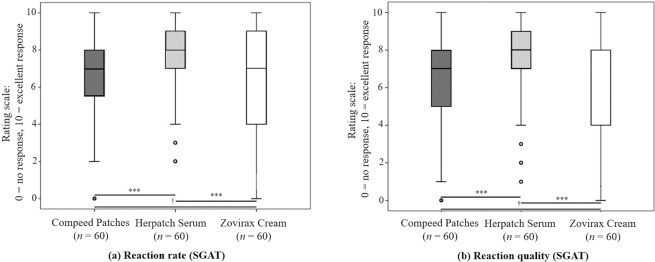
Median (25th and 75th percentile), minimum and maximum of the reaction rate (**a**) and reaction quality (**b**) of the product assessed by the subject (SGAT) at the end of therapy (day 10). Horizontal lines with “***” indicate significant differences between the products, and “†” indicates a lack of a statistically significant difference.

The median (25th and 75th percentile) reaction rate (SGAT) of Herpatch was 8.00 (7.00–9.00) and was significantly different (*p* = 0.025) from that of Compeed, with a median of 7.00 (5.25–8.00), and that of Zovirax, with a median of 7.00 (4.00–9.00). Additionally, the median (25th and 75th percentile) of the reaction quality (SGAT) was significantly different (*p* = 0.025) between Herpatch, with a median of 8.00 (7.00–9.00), and Compeed, with a median of 7.00 (5.00–8.00), as well as between Herpatch and Zovirax, with a median of 7.00 (4.00–8.00) (Fig. 4).

### Accompanying symptoms and product features

**Figure 5:**
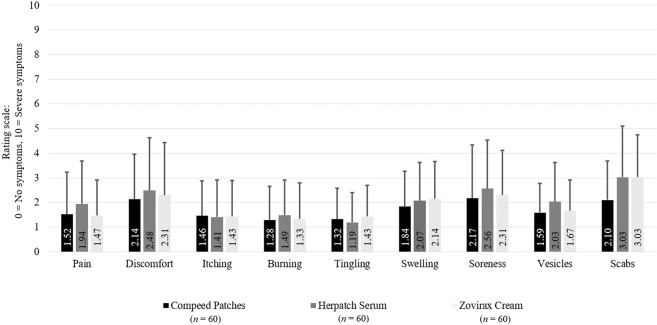
Subject-assessed accompanying symptoms.

All tested products were well tolerated and showed no serious adverse events. The development of symptoms showed a similar distribution in all three groups. While pain, discomfort, itching, burning, tingling, swelling, and blisters occurred primarily at the beginning of therapy, the appearance of soreness and crusts increased until the end of the treatment. All symptoms were rated on a scale from 0 to 10 (0 = no symptoms; 10 = severe symptoms) (Fig. 5).

**Figure 6:**
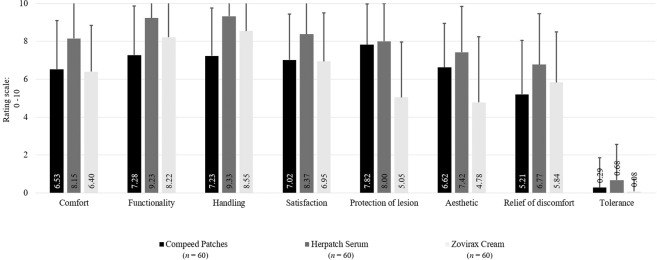
Subject-assessed product features during and at the end of study.

The assessment of product features was performed on a 0 to 10 scale. The highest ratings of lesion protection (0 = no protection; 10 = high protection), aesthetics (0 = poor aesthetics; 10 = high aesthetics), and relief of discomfort (0 = no relief; 10 = high relief) were achieved with Herpatch use. However, Herpatch led to the most intolerances (0 = tolerant; 10 = intolerant), such as dried lips or redness. The assessment of comfort (0 = no comfort; 10 = high comfort), functionality (0 = poor functionality; 10 = high functionality), handling (0 = hard handling; 10 = easy handling), and satisfaction (0 = no satisfaction; 10 = high satisfaction) were more in favour of Herpatch (Fig. 6).

## Discussion

Within this monocentre, randomized, assessor-blind study, the effectiveness of three different products in the treatment of HSL was evaluated in a total of 180 patients. In this study cohort, the sex ratio was in favour of females (81.67%). This can be explained by the higher prevalence of clinically manifested orofacial herpes simplex in females than in males^24–26^. In addition, the number of females working at the University of Witten/Herdecke is higher than the number of males. Due to the high number of participants in this single study, we included the patients as soon as HSL occurred, leading to a higher number of enrolled females. Comparable studies also did not consider the unbalanced proportion of male to female participants in the recruitment^22,27,28^. This can be considered a limitation of this study, but the advantage of this study is the high number of participants. The mean age of the participants is comparable to that of a study from Senti *et al*., with a mean of 32.6 years. They included patients from 18 to 50 years of age^29^. Clinical trials with a minimum age of 18 result in a higher mean age^30–32^. Participation lasted 10 days, which is equal to that of comparable studies^22,33,34^. Other studies have defined longer periods of observation of up to 22 days^35–37^. An extended observation span allows the monitoring of lesions with a longer healing time but might lead to a negative impact on the recruitment and compliance of patients. Treatment was initiated in the first 24 hours of the outbreak of the lesion. Within this time, the prodromal lesion turns into a vesicle^38^. Gross and Braun observed a vesicle on the first examination day in 88% of the patients. Because of the high prevalence of vesicles, this early lesion stage was considered the beginning of the evaluation of the healing time^33^.

The healing time of HSL lesions treated with Herpatch was comparable to that of lesions treated with Compeed and with Zovirax (primary endpoint). The differences among Herpatch, Compeed and Zovirax were small and not statistically significant. Herpatch contains several ingredients that provide perfect conditions for wound healing, while the Compeed semiocclusive hydrocolloid dressing promotes moist wound healing and reduces crusting^19,20^. Both products do not influence the viral replication cycle. Only Zovirax includes an antiviral agent (5% acyclovir)^17^. Application of this substance at an early prodromal stage leads to an inhibition of the viral replication of HSV-1 in the host cell^39^. The early prodromal stage generally presents with subclinical symptoms that are usually imperceptible to the patient. A delayed application of acyclovir at a later stage does not influence the healing time^36,40^.

The statistical analysis of the reaction rate and the reaction quality showed statistically significant differences between Herpatch and Compeed as well as between Herpatch and Zovirax, both in favour of Herpatch. Karlsmark *et al*. evaluated the same parameters without assessing statistically significant differences between Compeed and Zovirax^22^. Hydrocolloid wound dressings such as the Compeed patches promote re-epithelialization and reduce inflammatory responses^41,42^. However, in this clinical trial, the patch had to be removed several times due to the patch margins peeling off. This was caused particularly by liquids such as saliva, food or drinks or by facial expressions. The peeled-off margins led to discomfort and caused the patient to renew the patch. With every replacement, already formed crusts were torn off with the patch, causing pain while promoting primary wound healing. Accompanying symptoms such as pain, tingling, swelling, or burning were also assessed in comparable trials^43,44^. The duration and intensity of symptoms were rated and showed comparable distributions independent of the applied product. This evaluation is important to prove the effective impact of treatment on healing.

Creams for topical application, such as Zovirax, wear off quickly while eating or drinking. For this reason, topical creams do not persist for an adequate time on the lesion and need to be reapplied every three to four hours^45^. This leads to a higher rating of unsatisfaction, which was also shown in comparable clinical studies^46^. Skulason *et al*. also assessed the aesthetics of a transparent gel compared to that of a white cream. Most patients preferred the gel because of its high aesthetic qualities^3^. Subjects treating recurrent HSL with Herpatch indicated high comfort, good aesthetics, easy use of application and overall high satisfaction with the product.

In this study, three disparate materials were compared. Double blinding would have minimized the risk of bias^47^. Because of the use of three different materials, conducting a double-blinded study under true conditions would have been challenging. To ensure objective assessments, the clinician was blinded, and the evaluation of healing time by the clinician was determined as the primary endpoint. The reaction rate and reaction quality were defined as secondary endpoints.

Finally, it is important to mention that this clinical trial did not include a placebo group. The comparison of an antiviral substance to a placebo can prove the positive characteristics of the antiviral compound. While Zovirax leads to an active antiviral treatment, Herpatch and Compeed only contain ingredients for the relief of symptoms. The antiviral effect and benefits of acyclovir have been shown in several studies, where placebo led to severe symptoms while healing^48,49^. Nevertheless, a comparable study also observed no significant differences regarding the healing time between Zovirax and Compeed^22^. Additionally, studies using a placebo showed false results because of the placebo’s ingredients, leading to a comparable healing time as an acyclovir cream^36,50^. For these reasons, the inclusion of a placebo group was waived.

## Data Availability

The authors confirm that the data supporting the findings of this study are available within the article and its supporting materials.
